# Supplementary material for: Dissecting the Structural Dynamics of Authentic Cholesteryl Ester Transfer Protein for the Discovery of Potential Lead Compounds: A Theoretical Study
Source: Int J Mol Sci. 2023 Jul 31;24(15):12252. doi: 10.3390/ijms241512252 (PMC10418423; doi:10.3390/ijms241512252)
Supplement: Supplementary file 1 [file ijms-24-12252-s001.zip › ijms-2504151-supplementary.docx]

Article

Dissecting the structural dynamics of authentic cholesteryl ester transfer protein for the discovery of potential lead compounds: a theoretical study

Yizhen Zhao, Dongxiao Hao ^†^, Yifan Zhao, Shengli Zhang, Lei Zhang and Zhiwei Yang *

MOE Key Laboratory for Nonequilibrium Synthesis and Modulation of Condensed Matter, School of Physics, Xi’an Jiaotong University, Xi’an 710049, China; zyz9856@stu.xjtu.edu.cn (Y.Z.); dongxiaohao18@163.com (D.H.); zyfan0911@outlook.com (Y.Z.); zhangsl@xjtu.edu.cn (S.Z.); zhangleio@xjtu.edu.cn (L.Z.)

***** Correspondence: yzws-123@xjtu.edu.cn

^†^ Current Address: School of Electronics and Information Engineering, Ankang University,
Ankang 725000, China.


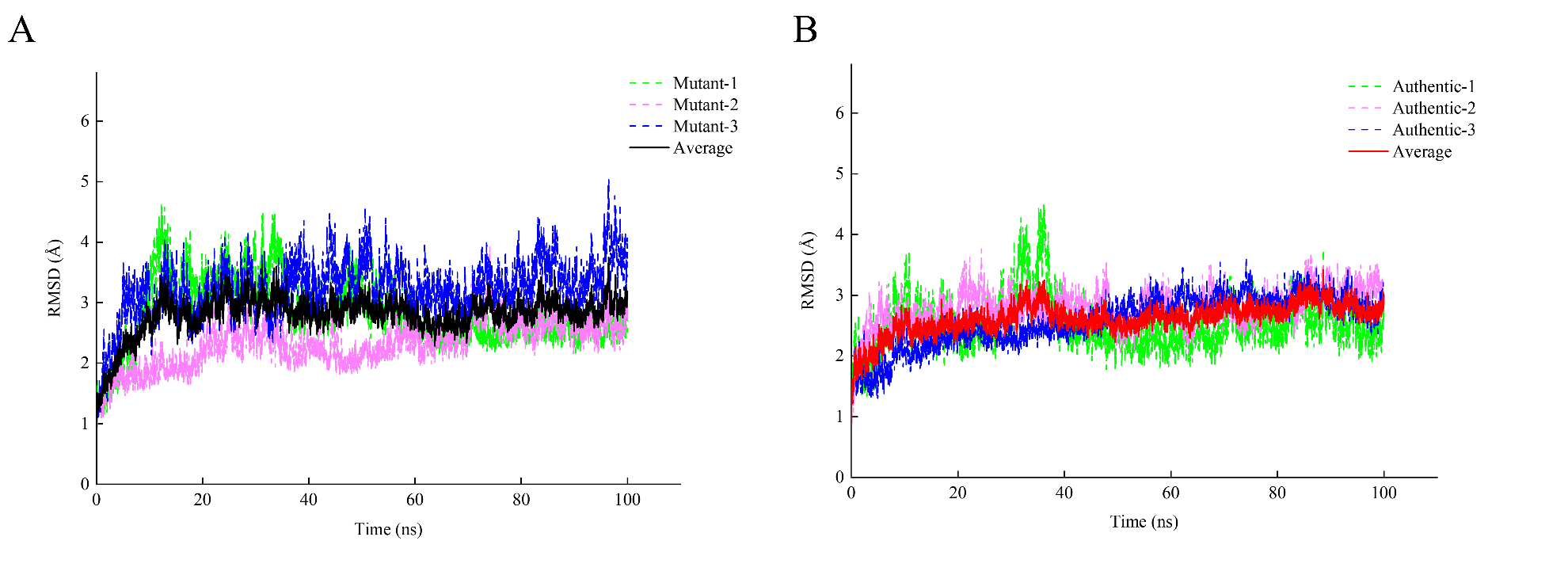


**Figure S1.** Variation of the backbone-atom root-mean-square deviations (RMSD) for (A) CETP^Mutant^ and (B) CETP^Authentic^ during MD simulations.


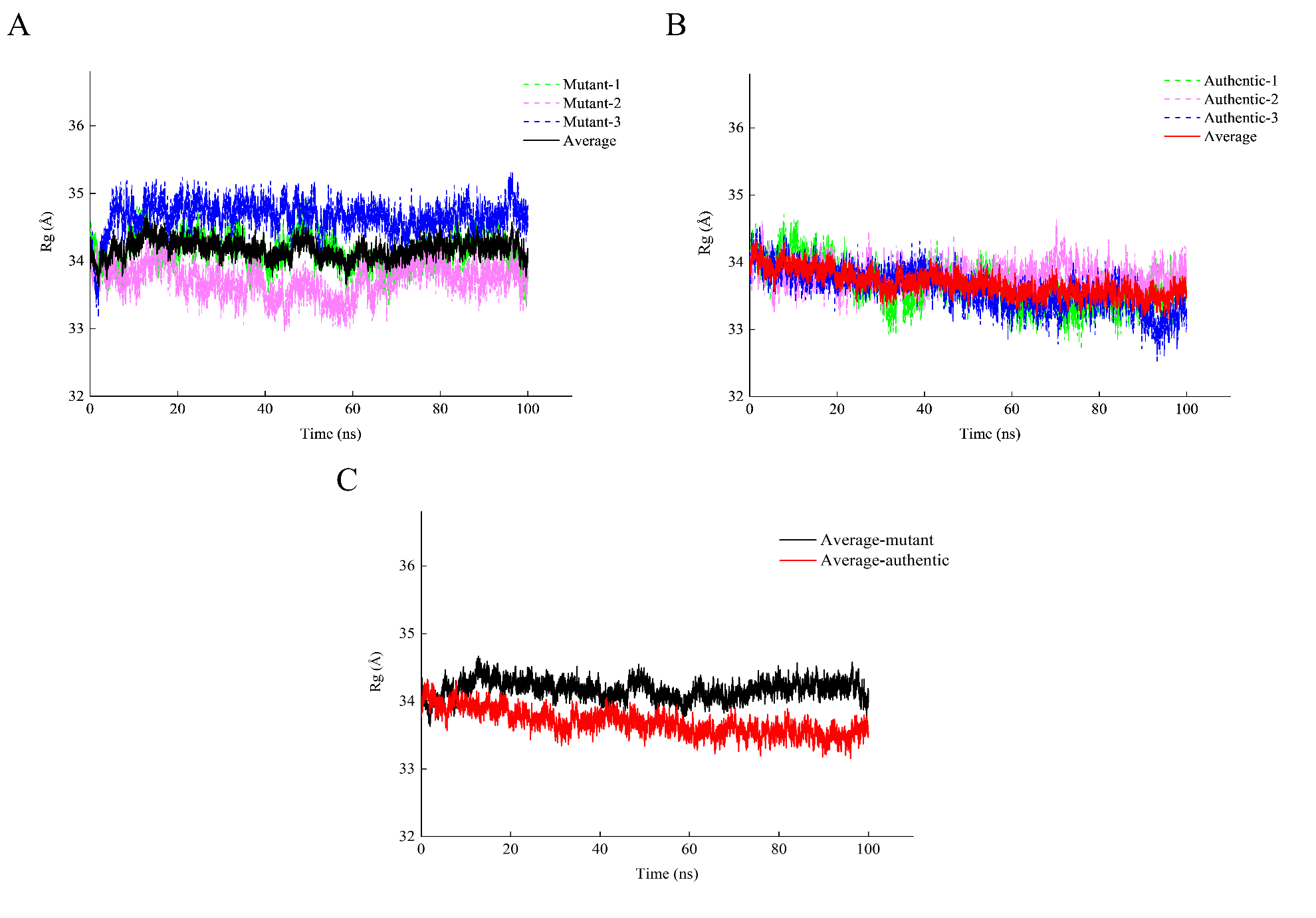


**Figure S2.** Variation of backbone radius of gyration (Rg) for (A) CETP^Mutant^, (B) CETP^Authentic^, as well as (C) contrast of Rg between CETP^Mutant^ and CETP^Authentic^ during MD simulations


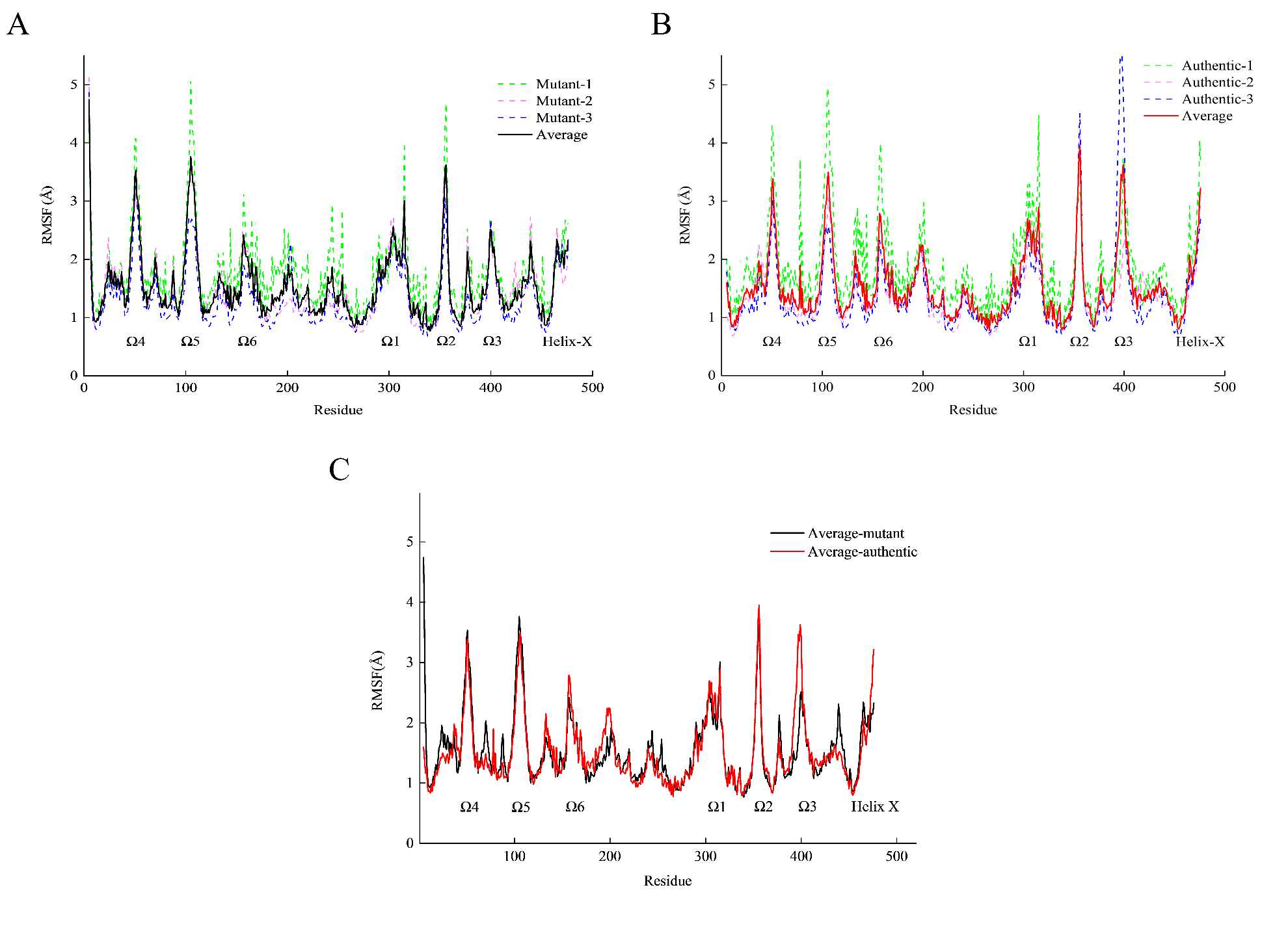


**Figure S3.** The root-mean-square fluctuations (RMSF) per residues of (A) CETP^Mutant^, (B) CETP^Authentic^, as well as (C) contrast of RMSF between CETP^Mutant^ and CETP^Authentic^. The initial 25 ns of these simulations are considered as a relaxation period and thus discarded.


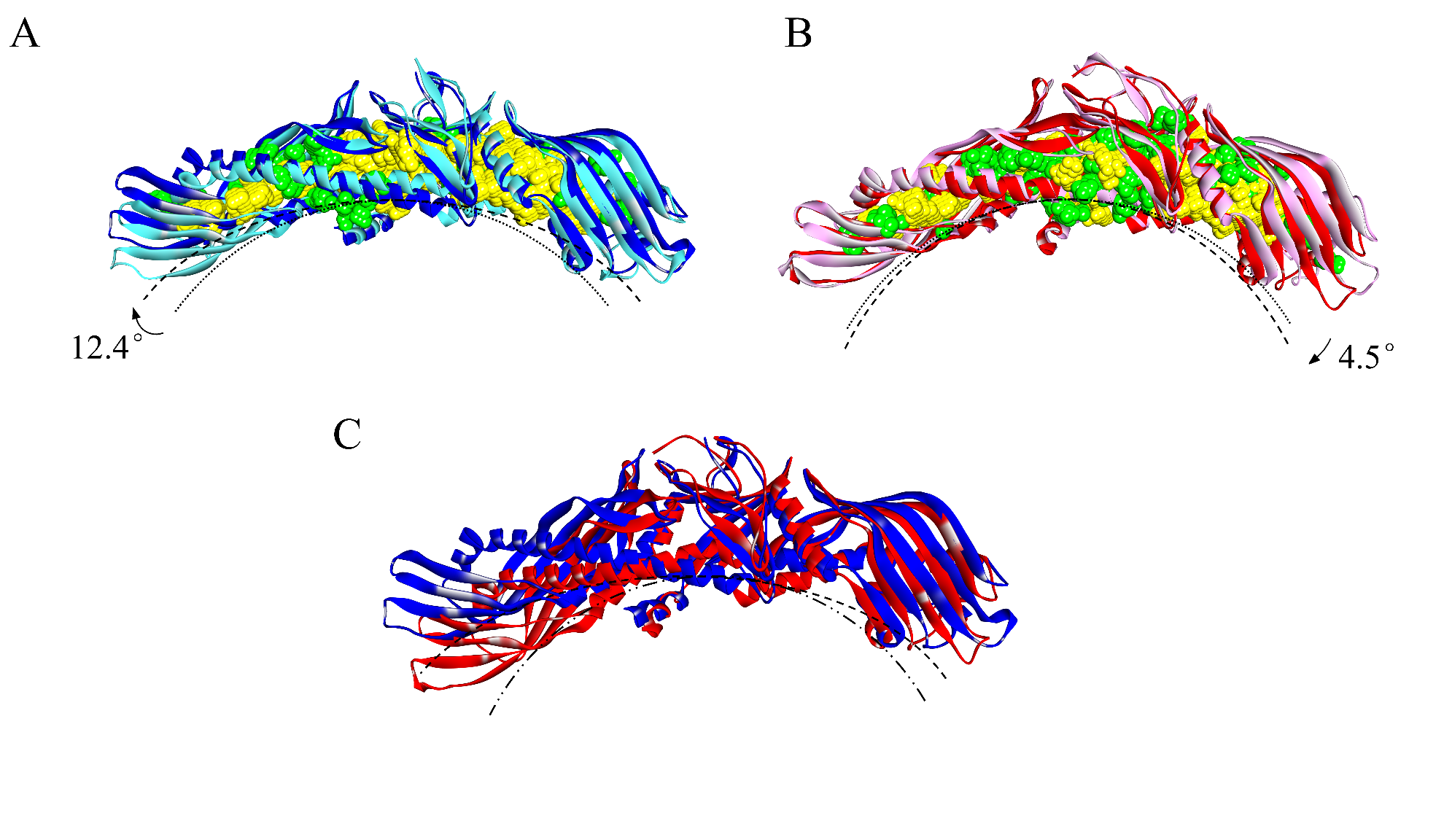


**Figure S4.** Structural contrast between the initiating and equilibrium conformations of CETP^Mutant^ (A, cyan and blue) and CETP^Authentic^ (B, pink and red), as well as contrast between two equilibrium conformations (C). Their internal cavities are shown in yellow and green, respectively; the stretch of protein is presented by the arrow.


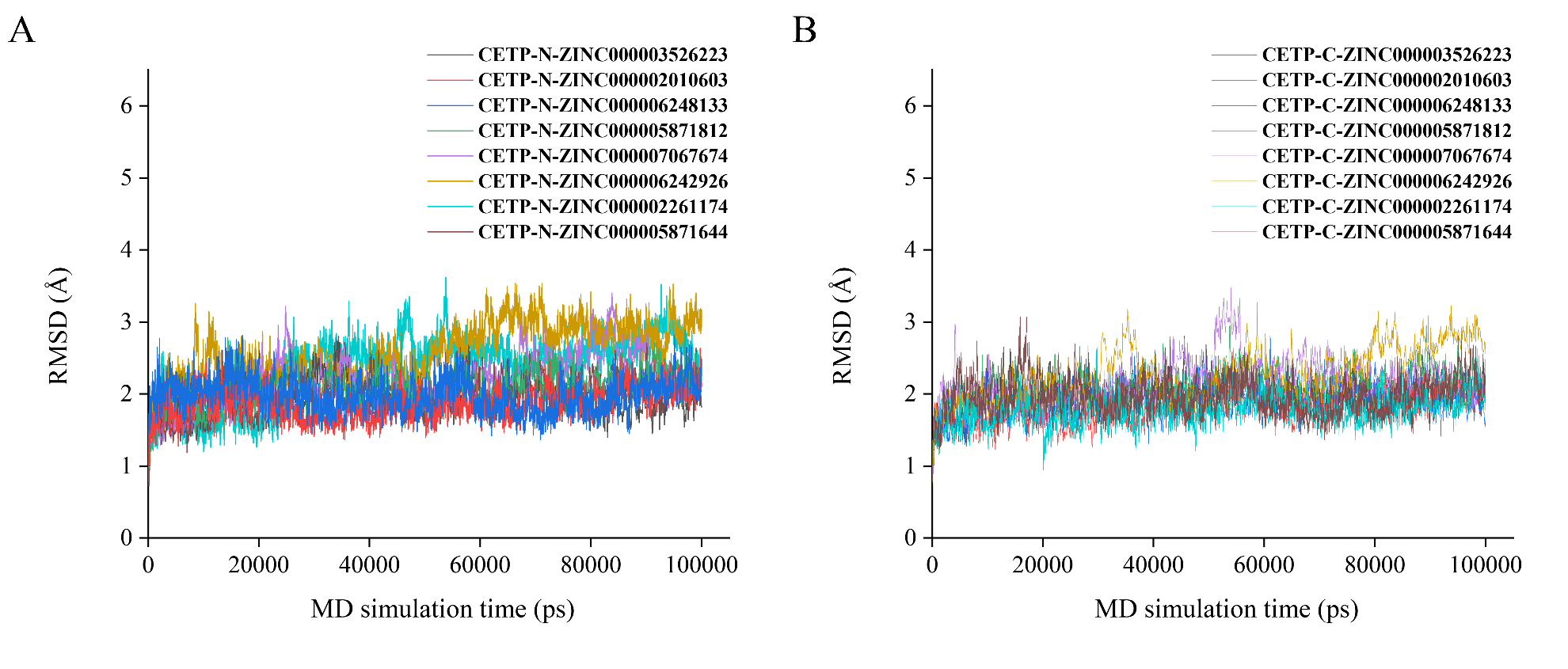


**Figure S5.** Variation of the backbone-atom root-mean-square deviations (RMSD) for (A) systems of ligand binding to the N-terminus of CETP^Authentic^ and (B) systems of ligand binding to the C-terminus of CETP^Authentic^ during MD simulations.


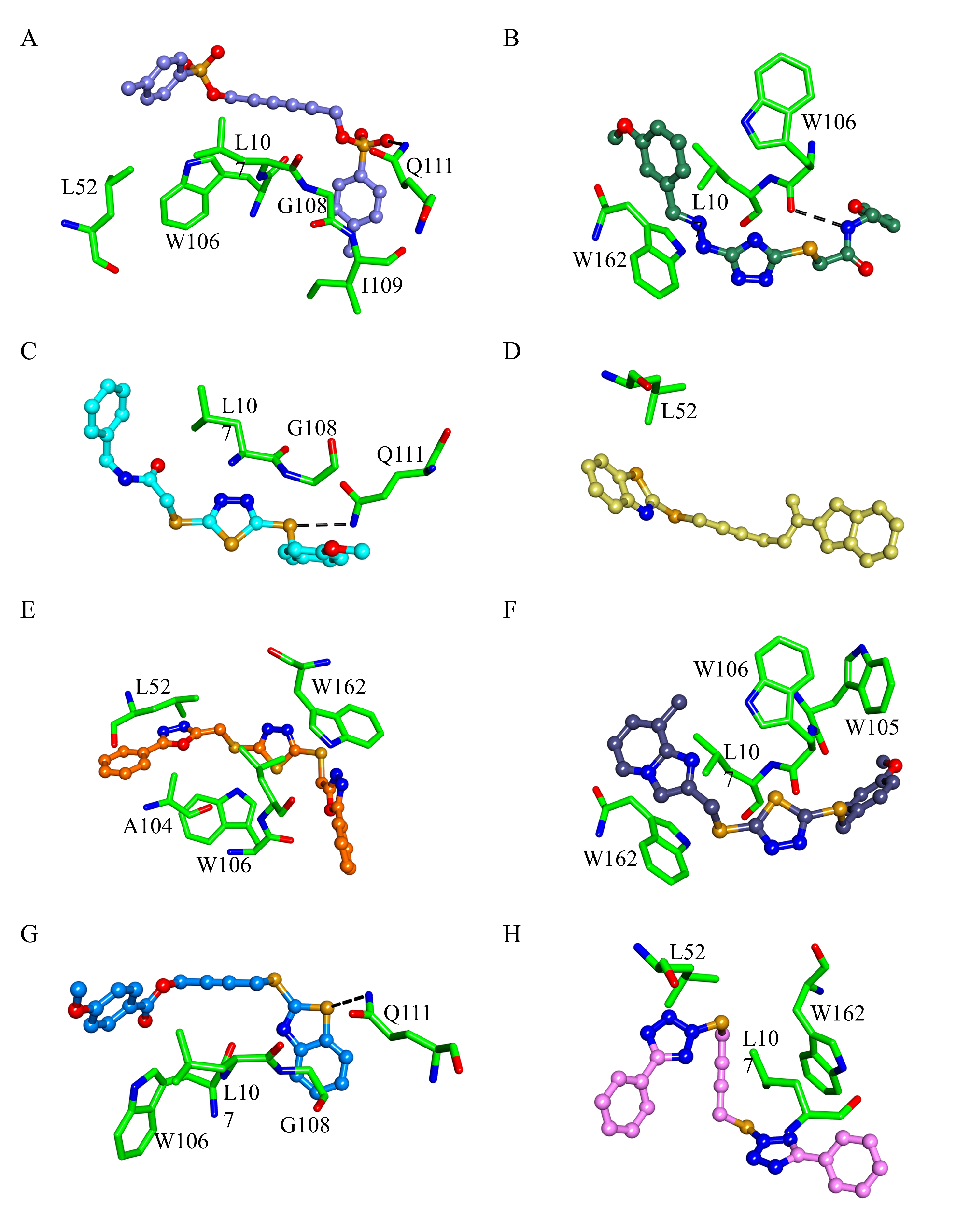


**Figure S6.** Detailed interaction analysis of CETP N-terminus and (A) ZINC000002010603, (B) ZINC000006248133, (C) ZINC000005871812, (D) ZINC000002261174, (E) ZINC000003526223, (F) ZINC000005871644, (G) ZINC000007067674 and (H) ZINC000006242926. The key residues are represented by stick models, and the important H-bonding interactions are labeled in the black lines.


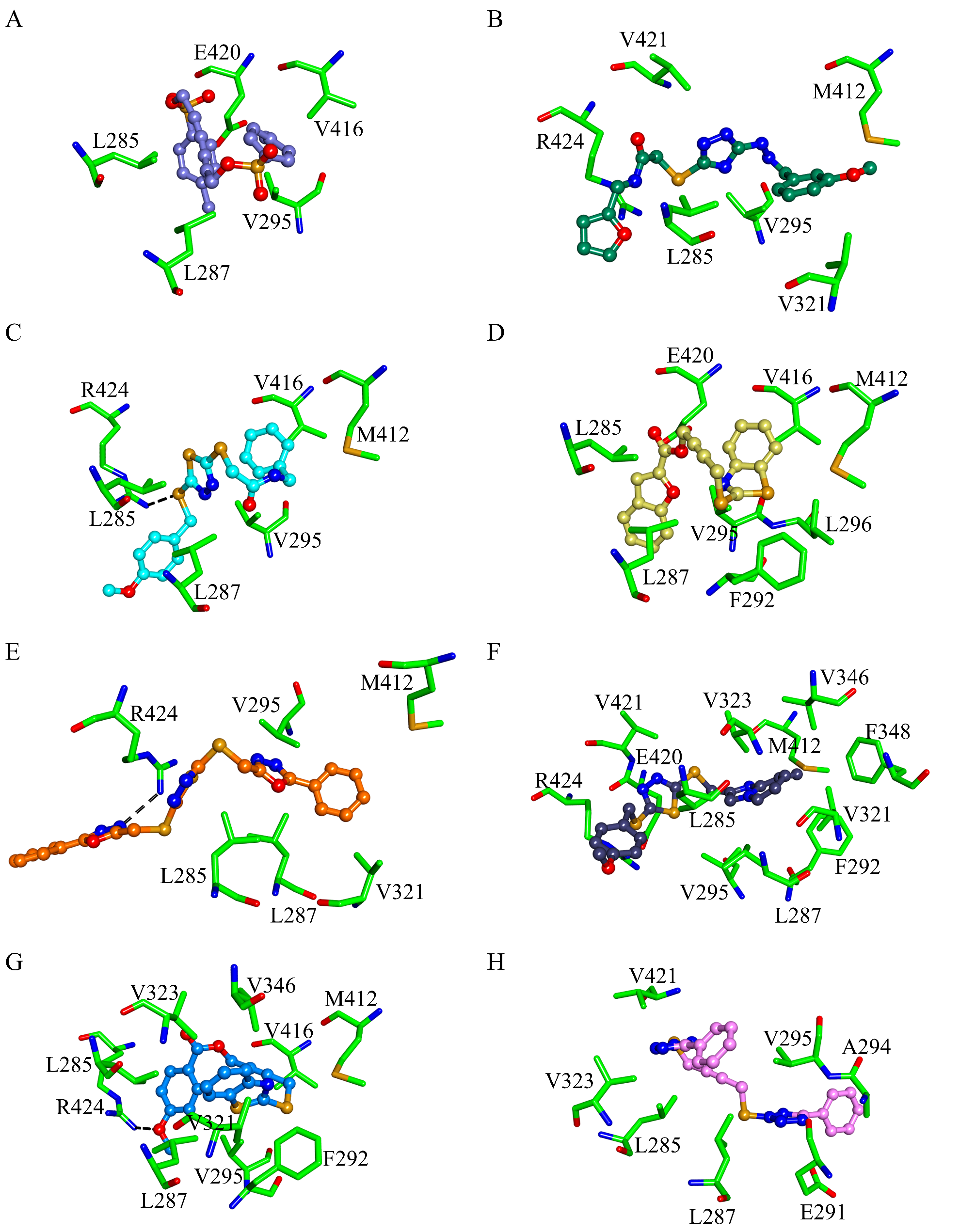


**Figure S7.** Detailed interaction analysis of CETP C-terminus and (A) ZINC000002010603, (B) ZINC000006248133, (C) ZINC000005871812, (D) ZINC000002261174, (E) ZINC000003526223, (F) ZINC000005871644, (G) ZINC000007067674 and (H) ZINC000006242926. The key residues are represented by stick models, and the important H-bonding interactions are labeled in the black lines.


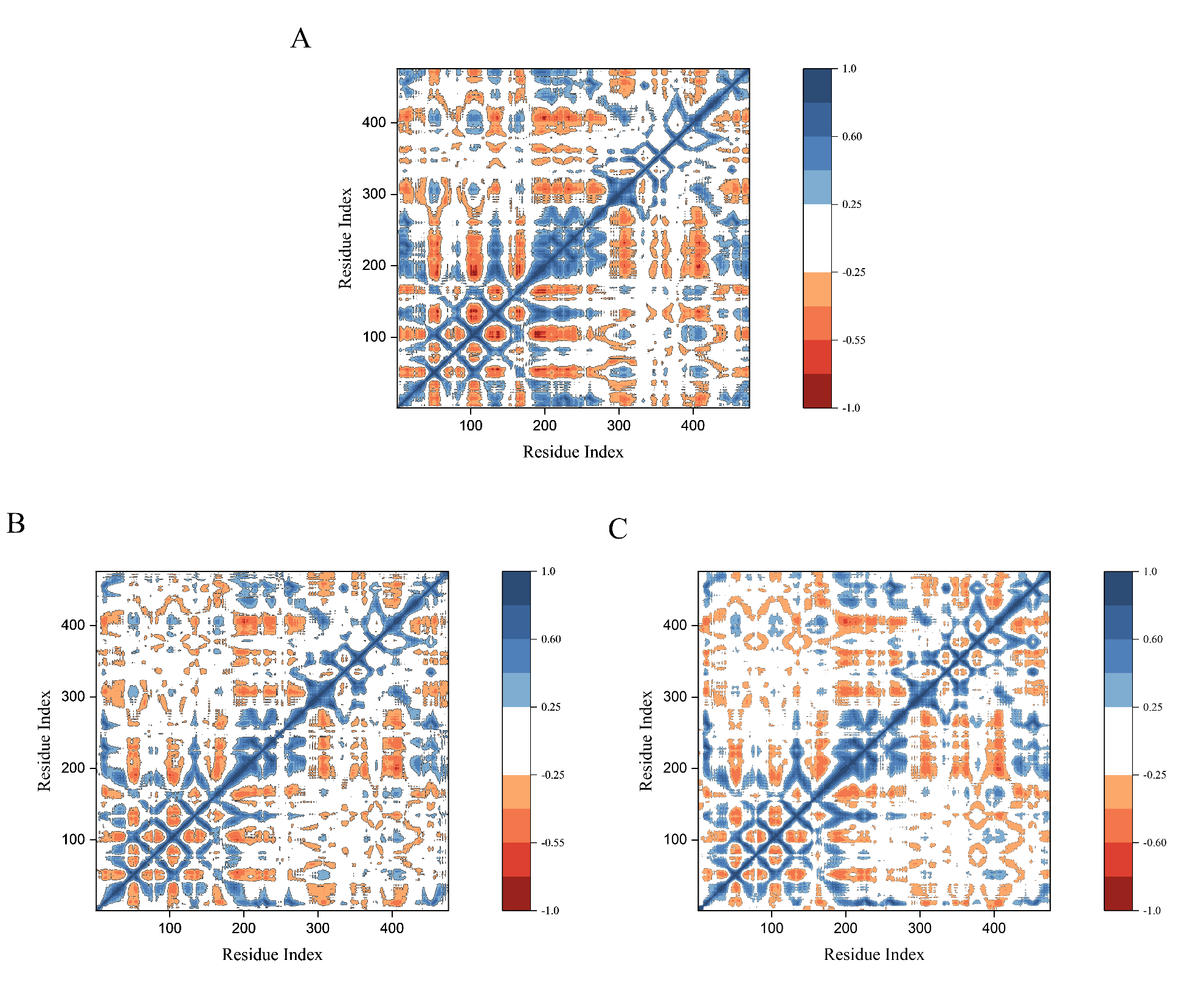


**Figure S8.** Dynamic cross-correlation maps for CETP in the (A) CETP^Authentic^, (B) CETP N-terminus-ZINC000006242926, and (C) CETP C-terminus-ZINC000006242926 simulations. Correlation values range from −1 to +1, the positive values (red) indicate that two residues are correlated and negative values (blue) indicate that they are anti-correlated.
